# Motor cortical processing is causally involved in object recognition

**DOI:** 10.1186/1471-2202-14-155

**Published:** 2013-12-14

**Authors:** Rebecca Decloe, Sukhvinder S Obhi

**Affiliations:** 1 Social Brain, Body & Action Lab, Department of Psychology & Centre for Cognitive Neuroscience, Wilfrid Laurier University, Waterloo, Ontario N2L 3C5, Canada

## Abstract

**Background:**

Motor activity during vicarious experience of actions is a widely reported and studied phenomenon, and motor system activity also accompanies observation of graspable objects in the absence of any actions. Such motor activity is thought to reflect simulation of the observed action, or preparation to interact with the object, respectively.

**Results:**

Here, in an initial exploratory study, we ask whether motor activity during observation of object directed actions is involved in processes related to recognition of the object after initial exposure. Single pulse Transcranial Magnetic Stimulation (TMS) was applied over the thumb representation of the motor cortex, or over the vertex, during observation of a model thumb typing on a cell-phone, and performance on a phone recognition task at the end of the trial was assessed. Disrupting motor processing over the thumb representation 100 ms after the onset of the typing video impaired the ability to recognize the phone in the recognition test, whereas there was no such effect for TMS applied over the vertex and no TMS trials. Furthermore, this effect only manifested for videos observed from the first person perspective. In an additional control condition, there was no evidence for any effects of TMS to the thumb representation or vertex when observing and recognizing non-action related shape stimuli.

**Conclusion:**

Overall, these data provide evidence that motor cortical processing during observation of object-directed actions from a first person perspective is *causally linked* to the formation of enduring representations of objects-of-action.

## Background

It is now well established that the primate brain is exquisitely tuned to process the behaviours of conspecifics (e.g [1]). Since the discovery of mirror neurons in the macaque monkey (di [2-4]), a wealth of evidence has accumulated to suggest the presence of a similar action execution-observation matching system in humans [5-7]. Transcranial magnetic stimulation (TMS) studies have consistently revealed activation of motor representations when an individual watches another agent performing an action, and recent studies have shown that the mere presentation of graspable objects is enough to increase motor cortical excitability [8-12]. Crucially, the activated representations are the same as would be active if the observer actually executed the action. This activation of common representations in observation and action conditions, suggests a low-level tendency to simulate the actions of other agents, and/or to prepare actions that are congruent with perceived objects (and in some cases, complementary to observed actions – see [13]).

To the extent that such motor activity occurs without the specific intent of the observer, it appears to be automatic [14]. There has been much theorizing about the role of motor activity during observation of actions and objects in social cognitive processes, with suggestions that it could be crucial for human abilities such as imitation, intention understanding, theory of mind and empathy [15-18]. Here in an exploratory study, we ask a different, but undoubtedly related question: Is motor activity during observation of object-directed actions involved in forming representations of objects-of-action for later recognition? We place this question within a broader context. Specifically, to the extent that motor cortical excitation during action observation is an automatic brain process, it is imperative to determine the full extent of its functional significance. We intuited that motor cortical excitation during observation might facilitate later action and/or object recognition, and we sought to test this idea by comparing recognition performance in conditions in which motor cortical processing was disrupted, with conditions in which motor cortical processing during observation was left intact. Again, here, the specific question relates to just one possible functional role of motor cortical activity during action observation – the ability to remember objects of action for subsequent recognition.

A critical component of processing, and ultimately understanding the actions of others, as well as learning how to perform actions ones self, is the ability to successfully encode and maintain a representation of actions *and the objects* of those actions. In social contexts, it is often necessary for an agent to remember the actions of another individual, and to remember the object that was the target of action as well. This ability to form and maintain memories of actions and objects of action seems fundamental to the ability to learn from observation, and to understand a similar goal oriented action at a later time. For example, if a child observes their mother pouring milk from a jug into a cup, it is useful for the child to form a representation not only for the action of pouring, but also for the jug and cup. In this way, representations of actions and objects are inextricably and functionally linked.

Here, we asked whether motor activity during observation of the actions of others might be critical for subsequent recognition of the object of an action. Participants engaged in an action observation task in which they observed a model on a computer monitor typing with a single thumb on a mobile phone, from either a first person, or a third person perspective. On some trials, 100 ms after the onset of the video, a single pulse of TMS was delivered either over the motor cortical representation of the abductor pollicis brevis (APB), a key muscle for thumb typing, or over the vertex, an area of the scalp overlying lower leg motor cortical representations not involved in thumb typing. In other trials, participants watched the same object oriented actions but no TMS was delivered. Immediately after the video, a mask appeared after-which participants were presented with a picture of a mobile phone and asked to judge whether the phone in the picture was the same or different to the one they had seen the model typing on earlier in the trial. The key prediction was that, if motor activity is important for remembering the object of action, participants should be impaired at recognizing the mobile phone on trials on which they received TMS over the APB representation, but not on no-TMS trials, or trials on which they received TMS over the vertex (which roughly overlies the leg representation of motor cortex). To ensure that any observed TMS effects were specific to action stimuli, we also included a control condition in which participants observed simple geometric shapes instead of object-directed actions.

As a secondary line of inquiry, we presented video clips from both first and third person perspectives, because previous TMS experiments on action observation have suggested a larger degree of motor facilitation when watching actions from a first person perspective [15,19]. Thus, we included separate blocks with videos shot from both perspectives to explore the potential role of motor activity during observation on recognition of the object of action.

## Methods

10 right-handed participants (six female, four male) between the ages of 18 and 21 participated in the study for course credit. All participants had normal or corrected to normal vision and the study was approved by the local ethics committee. All participants were screened for contraindications to TMS and provided written informed consent prior to participation. Wilfrid Laurier University Research Ethics Board is the body that approved the study

### Procedure

For the duration of the experiment, participants were seated with their right arm placed on a padded arm-rest in front of a computer monitor upon which all visual stimuli in the experiment were presented. The first part of the set-up involved localizing the scalp position overlying the APB muscle. This procedure was followed by the experiment proper, in which participants viewed action videos and control pictures (simple star shapes) and made same/different judgments about the stimuli present in the action videos and control pictures.

### TMS procedure

The site and stimulation intensity for application of TMS was determined by first locating the “hot-spot” for the APB muscle, and then selecting the stimulator output that produced MEPs of a criterion amplitude in 50% of trials. Specifically, motor evoked potentials (MEPs) were recorded via pairs of 8-mm surface electrodes placed in a belly-tendon arrangement over the right APB muscle, and a ground was placed on the ulnar styloid of participants’ right wrist. MEPs were recorded with a Biopac MP150 data acquisition system at 1 KHz, amplified (to 5 mV), filtered (band-pass 10–500 Hz) and sent to a computer running Acknowledge software. Vertex was located using the inion-nasion line and preauricular points at the posterior end of each zygomatic arch as landmarks. TMS was delivered through a figure-eight coil, held normal to the scalp and 45° to midline, with current flowing in a posterior-anterior direction over left primary motor cortex. Stimulation began at 70% of stimulator output and the coil was moved incrementally until the site eliciting the greatest MEP in the right APB muscle was identified. The optimal location was marked on a lycra swim cap worn by participants, and the coil was locked into a mechanical arm to fix it in position. Once the optimal location for MEPs was identified, stimulator output was lowered at 2% intervals until the minimum intensity capable of eliciting MEPs of approximately 1 mV peak-to-peak amplitude on 50% of TMS pulses had been identified [12,20]. This stimulation intensity was used for the remainder of the experiment. Coil position was monitored throughout the experiment using Brainsight v2.2.1 (Rogue Research, Montreal, Canada).

### Experimental procedure

Each participant was exposed to four blocks of trials, with half the participants being exposed to blocks involving a coil location over the APB first, followed by blocks involving a coil location over vertex, and the other half being exposed to blocks involving the vertex location first, followed by the APB location. Two of the four blocks contained videos shot from a first person perspective and two blocks contained videos shot from a third person perspective, but the order of blocks in terms of perspective was fixed across participants so that each participant first did a first person block, then a third person block, followed by a first person block, and finally, another third person block. Trials within each block were randomly presented and consisted of 18 TMS trials and 36 non-TMS trials. We also included non-action trials which served as control trials for later analysis, to ensure any effects were specific to stimuli containing actions. Thus, out of the 18 TMS trials, 12 involved action videos, and 6 involved non-action stimuli (simple star-shapes), and out of the 36 non-TMS trials, 24 contained actions and 12 contained simple non-action related shapes. In this way, in each block there were 54 trials, and TMS was delivered on 33% of trials.

**Figure 1:**
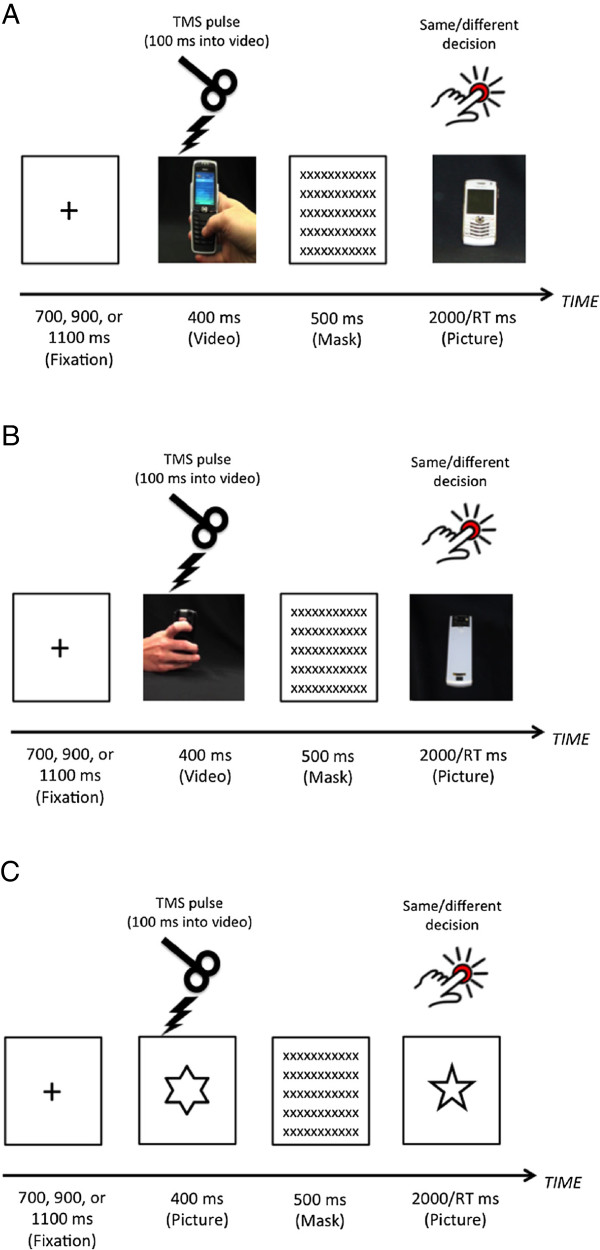
**Trial timelines for the different experimental conditions. A**. Example first person perspective trial. **B**. example of a third person perspective trial. **C**. example of a control trial in which a star shape was presented for 400 ms.

All experimental stimuli were presented on a 20 inch (Dell) LCD monitor. The sequence of events in each trial is depicted in Figure 1 and was as follows: A fixation cross was presented in the middle of the screen for 700, 900, or 1100 ms, followed by a video of a right hand thumb typing on a cell phone (or a photograph of a star shape in control trials) for 400 ms, followed by a mask for 500 ms. After the mask, a photograph of the phone (or a shape) was presented in the middle of the screen and participants were required to indicate whether the stimulus currently in the centre of the screen was the same or different from the one they had seen in the preceding video (or still picture in the case of non-action related shapes). Participants were asked to respond as quickly and accurately as possible and used their left middle finger to press the “s” key to indicate the stimulus was the same, or their left index finger to press the “d” key to indicate the stimulus was different. Importantly, left hand responses were used to minimize the effects of any kind of motor disruption of the right hand due to TMS over the left motor cortex. Stimulus presentation and reaction time data was recorded using Superlab v4.5 (Cedrus Corporation, San Pedro, CA, USA).

In action video clips, stimuli consisted of first or third person videos of a model thumb typing with their right thumb on a mobile phone and videos occupied almost the whole computer screen. The third person blocks were included as an additional question, but our expectation in these blocks was not specific as the videos themselves did not explicitly show a thumb movement. That is, participants simply observed the rear of a phone being held and a slight movement of the phone, without actually seeing a thumb. Six different cellular phone types were used, (selection was arbitrary, based on availability of phones from previous studies conducted in our lab). These comprised two different iPhones (Apple Corporation, Cupertino, CA, USA), three different models of Blackberry (RIM Ltd, Waterloo, ON, Canada), and one Nokia (Nokia corporation, Espoo, Finland). In non-action control trials, pictures of simple star shapes were presented.

TMS was delivered on a proportion of trials for all stimulus types and responses were made with the left hand to minimize potential motor disruption effects to the right hand caused by TMS delivered over left motor cortex.

Following the four experimental blocks, participants were asked if they were aware of the purpose of the experiment, and were then debriefed and informed of the hypotheses and purpose of the study.

## Results

The main dependent variables in the current study were speed (reaction time) and accuracy (percent correct) in the same/different judgment task. For the reaction time data, any responses that were outside 3 standard deviations from the participant’s own mean RT for a particular condition were excluded from the analyses. This procedure resulted in removal of less than 1% of trials overall. Video perspecitve was not treated as a factor, and therefore, each perspective was analyzed separately and the experiment comprised a 1 × 3 repeated measures design (factor: Condition, levels: TMS to APB, TMS to vertex, No TMS). Further, since there were non TMS trials with the coil positioned over APB and vertex, in the analysis these non TMS data were combined to produce a single “No TMS” condition.

### First person perspective videos

#### Accuracy analysis

**Figure 2:**
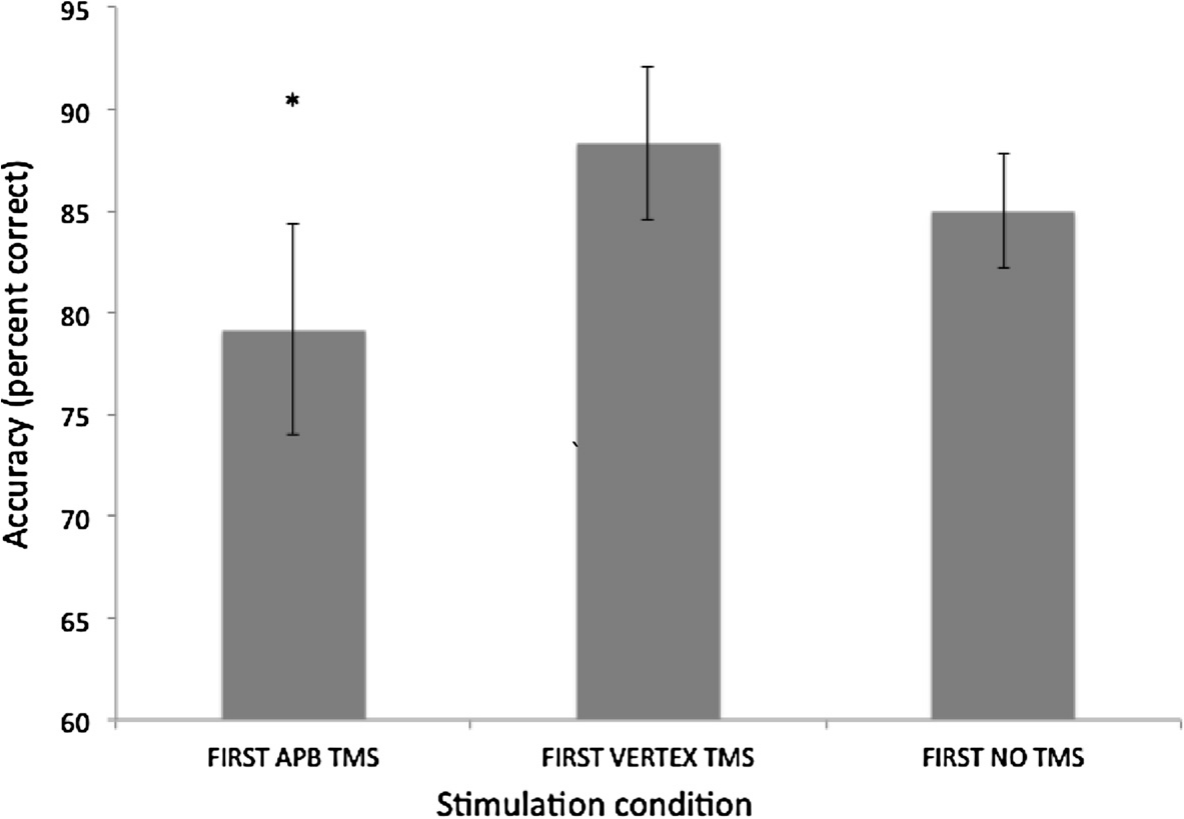
**Accuracy data for phone recognition task for first person perspective videos.** Note that recognition accuracy was significantly worse when TMS was applied over the APB representation during observation, compared to when TMS was applied over vertex, and no TMS conditions. Error bars are SEM, see text for statistics.

For each participant, accuracy scores (as percent correct) were entered into a 1 × 3 (condition: TMS to APB, TMS to vertex, No TMS) repeated measures ANOVA. This analysis revealed a significant main effect of condition (F (2, 18) = 4.831, p = .021). Planned (one-tailed) t-tests showed that TMS to APB resulted in significantly worse recognition accuracy compared to TMS to vertex (TMS to APB accuracy = 79%, TMS to vertex accuracy = 88%, t (9) = −2.905, p = .009), and no-TMS (no TMS accuracy = 85%, t (9) = −1.826, p = .051) – Figure 2. In contrast, TMS applied over vertex had no effect on recognition accuracy compared to the no-TMS condition (t (9) =1.300, p > .05). Thus, TMS applied over the APB representation impaired the ability to recognize the phone from the preceding video in the end of trial recognition test.

#### Reaction time analysis

**Figure 3:**
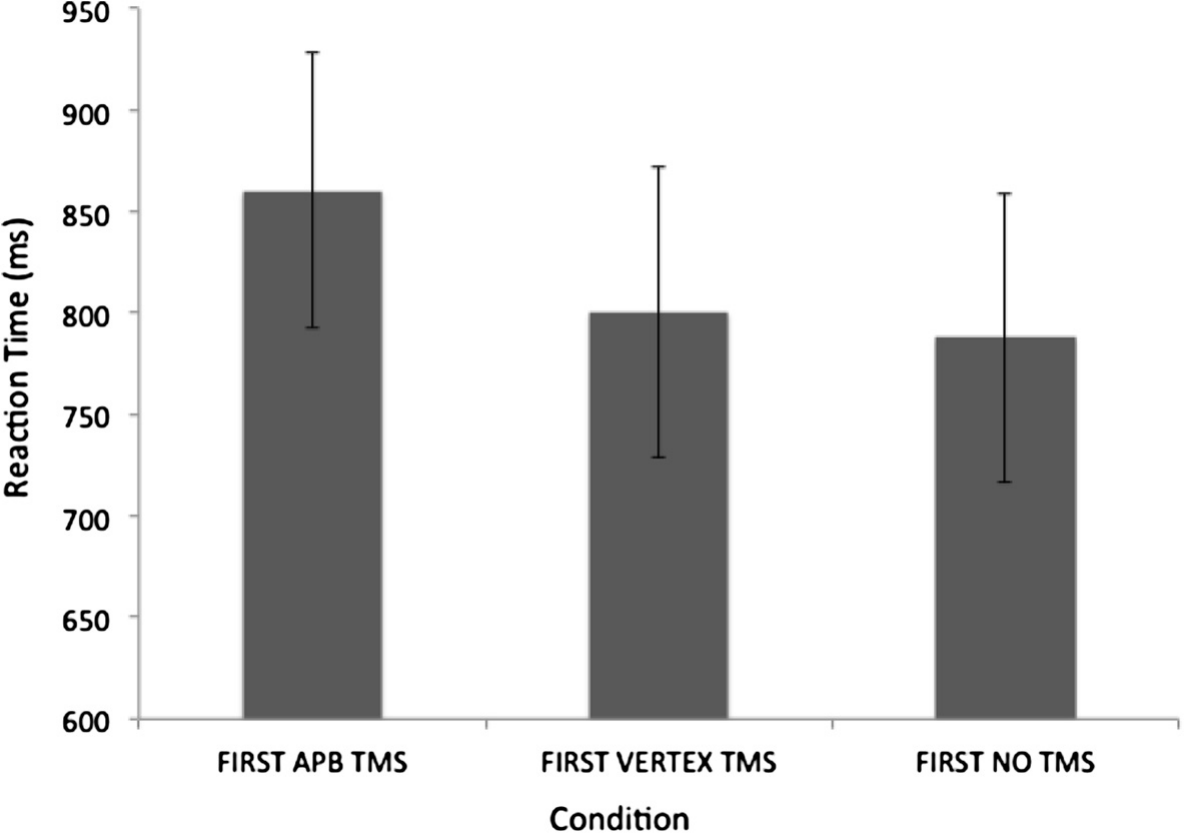
**Reaction time in the phone recognition test.** Note that RT was significantly slower when TMS had been applied over the APB representation during observation, compared to when TMS had been applied over the vertex and the no TMS condition. Error bars are SEM, see text for statistics.

RT data were entered into a 1 × 3 repeated measures ANOVA. This test revealed a significant main effect of condition (F (2,18) = 4.597, p = .024). Planned (one-tailed) t-tests showed that TMS to APB slowed RT in the recognition test more than TMS to vertex, although this result was at the border of statistical significance (TMS to APB RT = 860 ms, TMS to vertex RT = 801 ms, t (9) = 1.811, p = .052), and more than the no TMS condition (no TMS RT = 788 ms, t (9) = 3.530, p = .003) – Figure 3. In contrast, TMS applied over vertex had no effect on RT in the recognition task compared to the no TMS condition (t (9) = 0.608, p > .05). Thus, TMS to the APB slowed recognition performance compared to the other experimental conditions.

### Third person perspective videos

#### Accuracy analysis

**Figure 4:**
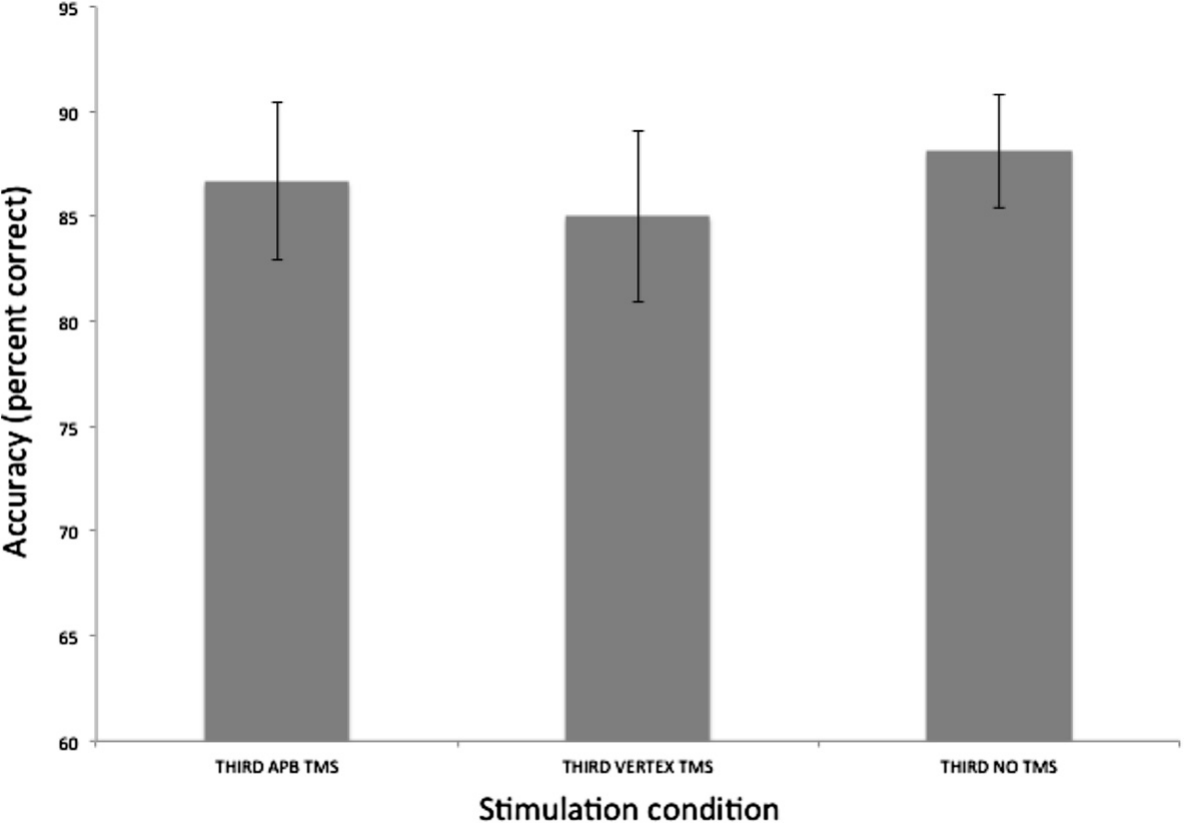
**Accuracy in recognition judgments in third person trials.** There was no difference between conditions. Error bars are SEM.

Accuracy data (as percent correct) were entered into a 1 × 3 (Condition: TMS to APB, TMS to vertex, no-TMS) repeated measures ANOVA. This test revealed no significant main effect of condition (F (2,18) = 0.400, p = .676) – Figure 4.

#### Reaction time analysis

**Figure 5:**
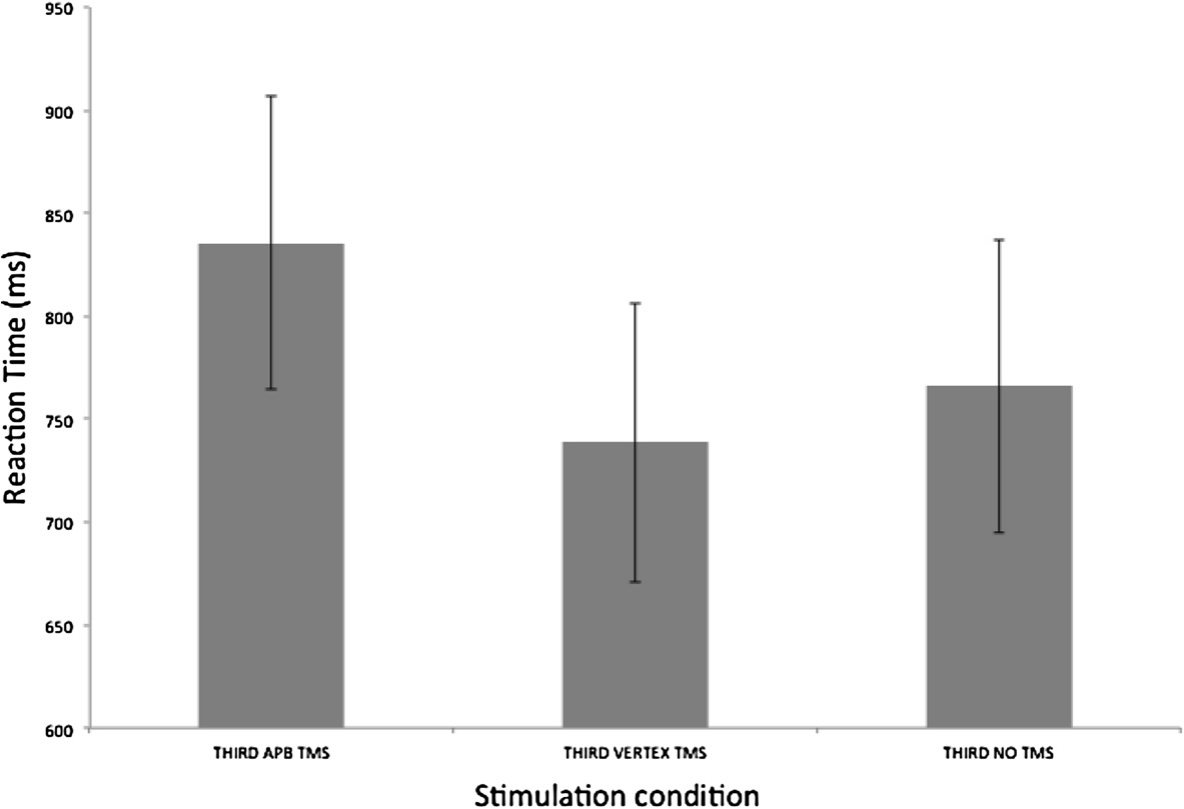
**Reaction time to make recognition judgment in third person trials.** There was no difference between conditions. Error bars are SEM.

RT data were entered into a 1 × 3 repeated measures ANOVA. This test revealed no significant main effect of condition (F (2,18) = 1.549, p = .240) – Figure 5.

Thus, for videos shot from a third person perspective, there was no effect of TMS applied over the APB on recognition accuracy or reaction time.

### Non-action stimuli - control shapes

As an additional analysis, we examined the data from the control shape trials separately. The star shape stimuli were used as a non-action recognition task to rule out the possibility that our TMS effects were general and not action specific. Thus shapes were always presented in a particular orientation and perspective from which the shape was viewed was not manipulated. Thus, perspective was not relevant for the shape accuracy and RT data, so accuracy data as percent correct were extracted from all blocks and subjected to a 1 × 3 (condition: TMS to APP, TMS to vertex, No TMS) repeated measures ANOVA. This test revealed no signifcant main effect of condition (F (2, 18) = 0.310, p = .737). RT data was subjected to the same analysis and again, there was no significant main effect of condition (F (2,18) = 1.668, p = .216).

Taken together, these results show that only TMS applied over the APB when videos are observed from a first person perspective produced impairments in recognition performance.

## Discussion

The aim of the current study was to determine whether motor processing during observation of object directed action, is related to subsequent object recognition. To investigate this question, we had participants watch a short video clip of a model thumb typing on a mobile phone, from either a first or third person perspective. On a portion of trials, we delivered a single TMS pulse 100 ms after video onset either at the scalp location overlying the APB muscle, which is involved in thumb typing, or over the vertex which roughly overlies the leg area of motor cortex and is therefore not involved in thumb typing. After watching the video, participants were presented with a photo of a mobile phone and had to respond as quickly and accurately as possible whether the phone was the same or different from the one they had seen in the preceding video. The results showed that TMS over the APB representation, produced significant decreases in accuracy for the object of action, when seen from a first person perspective only. There was no effect of TMS over the APB for recognition performance when videos shot from a third person perspective were observed. The same pattern of results emerged for the RT measure; when TMS was applied over the APB during observation of typing from a first person perspective, there was a slowing of recognition performance for the phone later in the trial, compared to when TMS was applied over the vertex, and no TMS trials.

The first question when considering our results is why there was an effect of TMS over the APB representation when participants were viewing actions from a first person perspective, but not when they were viewing actions from a third person perspective. Upon re-inspection of the video stimuli, a very obvious reason for this difference emerges (refer to picture of third person stimulus in Figure 1B): The thumb movement was not actually visible in the videos that were shot from a third person perspective. Therefore, whilst the first person video clearly showed a thumb moving (albeit for a fraction of a second), in the third person videos, participants only saw a slight movement of the phone held in the hand. The thumb was never visible, and the videos may have been too short to decipher what the person was doing with their phone in the third person trials. That is, higher level inferential processes may be necessary interpreting this kind of stimulus where a thumb movement cannot be observed, whereas the first person stimulus affords a clearer thumb movement and no higher order processing may be required. Thus, a crucial follow up experiment is to construct videos in which the third person perspective is shot in such a way that the thumb is still visible. This would allow us to determine if the effect is truly only apparent for first person videos, or whether one simply has to be able to see the thumb moving for the effect to arise. Our prediction is that the latter possibility is the case, but again, it remains for a future study to confirm this. For the rest of this section, we focus on the first person results, which in themselves are intriguing.

These results suggest that, in addition to the commonly held notion that motor processing during observation of actions is crucial for social cognitive functions such as action understanding, theory of mind and empathizing, it is also causally involved in the ability to recognize objects of action after initial exposure to those objects within an action context. We thus provide some of the first evidence for a basic cognitive role for motor processing in forming representations for subsequent object recognition, at least when those objects are embedded in action contexts.

It is worth noting that we took extra care to minimize any potential effects of the TMS pulse on motor responding, by requiring participants to respond with the hand ipsilateral to the side of TMS delivery (i.e., the left hand). Therefore, the idea that responses were slowed due to TMS effects on motor responding is very unlikely. Furthermore, TMS effects on motor responding are effectively ruled out because there were no differences in RT or accuracy for the shape stimulus, even when TMS was applied over the APB representation. Another possibility is that TMS to APB caused facial muscle twitches/eye-blinks to a greater extent than TMS to vertex, owing to the more lateral coil positioning for APB stimulation. However, given that this would have affected basic vision of the stimulus, and the fact that we did not observe any detrimental effect of APB TMS in the shape recognition trials, this possibility is unlikely to explain our results. Finally, it is possible that the TMS to APB actually faciliated participants to focus on the thumb, hence taking attention away from the object. Whilst this possibility could be relevant, very recent new data from our lab has shown a similar effect for recognition of the effector after watching a video involving squeezing a rubber ball between the index finger and thumb (unpublished data, in preparation). This suggests that it is not distraction away from the object by TMS to APB that is driving the effects we report in the current paper.

The ability to form successful memory for actions and the objects of those actions is a key requirement for learning from observation and understanding basic meaningful action-effect relationships, among other things. Recent work has shown that TMS elicited MEPs are facilitated 120 ms after participants view real 3-D graspable objects [10]. In line with ideas from Gibsonian perception, Franca et al. [10] suggested that exposure to objects affords actions toward those objects, and that this action planning is captured in specific facilitation of MEPs recorded from muscles that would be involved when interacting with the object [21]. Our work extends these new findings by demonstrating that, above and beyond motor facilitation during object processing, this motor activity appears to be *causally linked* to the ability to recognize those objects in the future. Furthermore, we demonstrate that the involvement of motor cortex is important at 100 ms post stimulus onset, which is quite similar to the 120 ms effect reported by Franca et al. [10]. Future work must address whether the 100 ms time point is crucial or whether other latencies of TMS delivery produce similar effects.

It has been previously reported that there is an early non-specific facilitation of motor cortex excitability during action observation that is detectable 90 ms after onset of the action stimulus [20]. The speed of this increase in motor cortical excitability suggests that there may exist a direct, perhaps thalamo-cotical route, that does not depend on input from visual brain areas ([20]; Thierry et al., 2008; [22]). The non-specific early facilitation found at 90 ms post stimulus onset by LePage et al. [20] complements the more specific facilitation effect for graspable objecs found 120 ms post-stimulus onset by Franca et al. [10], and the results from the current study fit well with the idea that motor cortex is activated realtively quickly after stimulus onset.

A novel contribution from the current work is the idea that motor processing during action observation is not only useful for potentially accessing goals and intentions linked to specific action representations ([4]; [23]), but also for more basic ‘cognitive’ processing of movement related stimuli. Whereas other work has alluded to the role of motor cortex in the processing of actions for social cognitive abilities such as understanding the mental states of others [6], our work firmly identifies motor cortex as an essential component of a memory network, that is crucial for laying down memories for the objects of action. Previous work has shown that in humans, mirror type activity occurs for many observed goal-directed actions [4,24-26]. Moreover, the recent study by Franca et al. [10] suggests that objects, when viewed in the absence of actions, also result in motor cortex excitation. In view of these findings, there are multiple possibilities regarding what aspect of the stimuli used in the current experiment were critical for our effects For example, it could be that motor cortex activity is critical in processing the object itself, independent of the action performed with the object (i.e., possibly based on input from canonical neurons), or that activity is contingent on a combination of action and object.

On a related note, in the present study, we did not test recognition of the specific effector or specific movements so it is impossible for us to claim with any degree of confidence, based on the current data, that motor activity is also involved in remembering specific actions. However, it is noteworthy that, in a more recent study from our lab (in preparation) we have found a similar effect of TMS on effector recognition, again when videos consisted of object oriented actions. Further experiments will examine these findings in more detail to determine exactly what aspect (s) of a stimulus display the motor cortex activation is driven by.

## Conclusion

We provide some of the first evidence that disrupting motor processing during exposure to object oriented actions, impairs subsequent recognition of the object of action. This finding raises the interesting idea that motor activity during exposure to object oriented actions might be *causally linked* to the ability to remember objects of actions. The idea that motor cortical processing is causal for memory for objects is a relatively novel suggestion, and we believe it opens up many important questions for future research.

## Competing interests

We declare that there are no financial or non-financial competing interests associated with this work.

## Authors’ contributions

SSO & RD designed the experiment. RD conducted the experiment. RD & SSO analysed the data. SSO & RD wrote the paper. Both authors approve the final manuscript.
